# The use of TikTok among children and adolescents with Eating Disorders: experience in a third-level public Italian center during the SARS-CoV-2 pandemic

**DOI:** 10.1186/s13052-022-01308-4

**Published:** 2022-07-30

**Authors:** Jacopo Pruccoli, Marta De Rosa, Lucia Chiasso, Annalisa Perrone, Antonia Parmeggiani

**Affiliations:** 1 IRCCS Istituto Delle Scienze Neurologiche Di Bologna, Centro Regionale Per I Disturbi Della Nutrizione E Dell’Alimentazione in Età Evolutiva, U.O. Neuropsichiatria Dell’Età Pediatrica, Bologna, Italy; 2 Dipartimento Di Scienze Mediche E Chirurgiche, University of Bologna, Via Massarenti 9, 40138 Bologna, Italy

**Keywords:** TikTok, Internet, Social media, Eating disorders, Body-Shaming, Children, Adolescents

## Abstract

**Background:**

Recent research has documented the potential associations existing between the use of social media (SM) and the occurrence/development and treatment of Eating Disorders (ED). However, the literature directly addressing the use of SM TikTok among children and adolescents with ED is still scarce.

**Methods:**

In January–February 2021, during the second Italian national lockdown due to the SARS-CoV-2 pandemic, an anonymous paper survey was conducted in an Italian third-level center for ED in childhood and adolescence. Demographics, frequency of use of TikTok, frequently viewed topics and hashtags, experienced body-shaming, as well as the use of TikTok (active search, use of proposed contents) and perceived influences of this SM on eating attitudes and self-esteem were assessed. Groups of patients with different perceived SM-induced effects were compared to determine the frequency of their interaction with 3 specific contents (diet, Pro-Anorexia Nervosa (pro-Ana) and pro-ED recovery).

**Results:**

Seventy-eight patients (93.6% females, mean age 14.5 ± 2.1 years) were enrolled in the study. For 62.8%, TikTok represented the main SM, used for 1.4 ± 1.0 h/day, with diet (21.8%) as the most frequently used topic category. Pro-Ana and pro-ED recovery contents (“#foryou” and “#edrecovery” as the most frequent, respectively) were both actively searched by patients and proposed by the SM in a significant number of cases. For 59.0%, using TikTok reduced self-esteem, while 26.9% reported TikTok-related significant changes in their daily lives, and 3.8% reported experiences of body-shaming. Patients describing a negative effect of TikTok on their self-esteem more frequently searched and browsed “diet” (*p* = 0.007) and pro-ED recovery (*p* = 0.007) contents. Positive qualitative feedback on the SM was also reported.

**Conclusions:**

This study documents the use of the SM TikTok among children and adolescents with ED. Individuals with a perceived negative effect of this SM on their self-esteem may show greater interaction with specific content. Further studies are needed to investigate the psychopathological factors influencing the relationship between ED and the use of SM.

## Introduction

Eating Disorders (ED) represent a group of psychiatric disorders characterized by disturbance of eating behaviors, which results in altered food consumption and impairments in physical health and psychosocial functioning [1].

The clinical burden of ED is frequently worsened by a series of mental or physical comorbid conditions, such as Mood Disorders, Personality Disorders, Autism Spectrum Disorder, and cardiovascular problems [2–5]. According to recent studies and clinical guidelines addressing ED in the developmental age, ED treatments may include nutritional interventions, family-based treatments, cognitive behavioral therapy, adolescent-focused psychotherapy, and psychotropic drugs [6–8].

Current research suggests a role that multiple and different factors play a role in the pathogenesis of ED, including genetic, neurodevelopmental, environmental and cultural factors [9]. Specifically, multifactorial hypotheses on the pathogenesis of ED distinguish among predisposing (genetic, biological, individual and family psychological, social), precipitating (abuse, illness, divorce) and maintenance (hormonal and neurotransmission imbalances) factors [10, 11]. In recent years, the role of Internet use in the pathogenesis of ED has been repeatedly studied [12, 13].

Two new terms have become part of the common language of the Web: pro-Ana and pro-Mia. Pro-Ana, which stands for pro-Anorexia, means “promoting Anorexia Nervosa” (AN). This may refer to websites, blogs, or communities that enhance AN and provide users with advice on how to achieve AN and its commonly-referred features. Similarly, pro-Mia, which stands for pro-Bulimia, means “promoting Bulimia Nervosa” (BN) [13, 14]. Pro-Ana and pro-Mia (pro-ED) communities promote ED as a lifestyle choice rather than as disorders that ought to be treated [15–18]. These trends – in fact “movements” with followers and devotees – have massively evolved since their first appearance in and as websites in the 1990s [19] on account of the radical change brought about by “highly interactive” social media (SM) communication dynamics. Sites have changed from providing a static and heavily moderated context to being a more visual and considerable [1] less hierarchical environment, which is more easily accessible and open due to the high number of public accounts and thus, more difficult to moderate due to the large volume of cross-tagged images and related hashtags [20].

The focus of this investigation is the SM TikTok, which was launched in 2016 and is one of the fastest-growing mobile applications with an estimated 800 million active users, the majority of whom are children and teenagers [21, 22]. This application is based on the sharing of brief videos associated with captions and several hashtags. Its remarkable peculiarity is that it is a completely algorithm-driven and content-oriented platform; so, each user will automatically receive personalized content on their main feed based on user interaction (i.e., like, dislike, but also video completion rate), video information (e.g., hashtags, captions or sounds) and account settings. Moreover, unlike other SMs, TikTok users receive personalized content from anyone on the platform rather than just from the accounts that they follow [23, 24].

Given the platform’s architecture and the variety of cross-linked hashtags, users who may be watching videos on weight loss or calories checks may soon receive a greater variety of eating-related information in their main feed, from “thinspiration” contents, which cover more mainstream dieting practices, to clearly “pro-Ana” ones [25].

A similar dynamic has been descripted for the phenomenon known as “challenges” [26]. Challenges bring many TikTok users to make videos of their attempts to reproduce the same specific trending tasks. Some of these challenges revolve around ED-related topics. Examples include the “#A4waistchallenge”, where people demonstrate that the size of their waistline is less than the width of an A4 sheet of paper placed vertically, or the “#headphonechallenge”, based on measuring waists using headphone wires [27, 28]. In a socio-cultural context already characterized by an increasingly blurred line between pro-ED contents and mainstream espousals of thinness [29, 30], TikTok challenges facilitate and increase the exposure of a growing number of users to all kinds of ED-related problematic content, which adds to the impressive adaptability of the app – through, for instance, the simple creation of new hashtags via the misspelling of previously banned ones – to evade strict oversight [31–33].

At the same time, due to the more open and public configuration of this application and the changed approach of new generations of users, who tend to share more openly and are more committed to raising awareness about and normalizing mental health issues [34], TikTok has simultaneously proved to be a thriving opportunity to create an easily accessible digital space for the positive content of such hashtag communities as “pro-EDrecovery”, “raisingEdawareness” and others [35, 36]. Clustering under the same hashtag, the users of these communities share their experiences and provide advice and support to one another by sharing their recovery processes, struggles and personal stories. They assert themselves and others by challenging the emphasis society and culture place upon being thin. They do so, for example, by countering those trends which praise thinness, by using the same hashtags to spread, instead, body-positivity messages (see #dontjudgechallenge #bodyodychallenge) [37]. It is thus possible to categorize materials and hashtags relating to diet as a general concept (food, its preparation and relationship with the body), pro-Ana (promoting eating disorder-related behaviors and mindsets), and pro-ED recovery contents (focusing on spreading awareness about ED and encouraging people with to continue their treatment path).

In light of all the above, a pressing need exists to understand the complex nature and conflicting characteristics of SM usage, and to fully explore how a relationship with SM, whether actively sought or accepted through passive exposure, can influence a sensitive population group such as our young and adolescent patients with regard to body image concerns and eating disturbances. This paper describes the use of TikTok in a group of children and adolescents referring to a third-level Italian center for ED during the SARS-CoV-2 (COVID-19) pandemic, with the goal of identifying general patterns in the use of TikTok by young people with ED. This study is the first survey conducted in Italy on the use of TikTok among patients with ED in developmental age. Our working hypothesis is that the analysis of the personal views of individuals with ED on TikTok may provide new insights into the potential risks and benefits of SM use in mental health.

## Methods

### Participants

A paper survey was administered during a 3-week period uninterruptedly, from January to February 2021. The survey was conducted at a third level Regional Center for Feeding and Eating Disorders in children and adolescents of the Institute of Neurological Sciences of Bologna, Italy. Respondents were recruited from outpatients and inpatients services for young and adolescent patients with Feeding and Eating Disorders. The authors proposed the survey to every patient, who frequented the center during the selected period. Every patient was asked to complete the survey only once during the selected period. Reasons for refusal to join the survey were noted and categorized as “lack of intention to disclose personal information”, “lack of use/insufficient knowledge of SM”, “other”.

### Measures

Due to the absence of previous surveys on TikTok use in the medical literature, we developed an anonymous questionnaire to survey our patients. The questionnaire included items that we drew from the medical literature on the general use of the Internet and of social networking sites among children and adolescents with ED [13, 20, 35, 38]. Two authors (J.P., M.D.R.) produced an initial draft of the questionnaire, which included 45 items, with a partial overlap between several of them. The authors of the questionnaire – a physician and a medical student – are both TikTok users and practicing in the field of ED in young age. To produce a handy paper survey that could be administered during routine clinical evaluations, the authors retained only 30 items concerning the use of TikTok among patients with ED. The remaining 30 items were independently evaluated by 2 reviewing authors (A.Pe., L.C.), who identified 14 items for inclusion in the final questionnaire. A third reviewing author (A.Pa.) assessed the final draft and resolved disputes between the two reviewers. All 3 reviewers are physicians working in the field of ED in young age. As a result, a total of 14 unique items were retained and included in the final draft of the questionnaire.

To explore diverse aspects of the use of TikTok among young patients with ED, the survey included different types of items. Demographic questions were restricted to age and sex, as the survey remained anonymous, and no personal or clinical data were collected from hospital documentations. Closed-ended questions (yes/no) were included; they regarded the use of TikTok, the influence of TikTok on a patient’s eating attitudes, participation in “challenges”, experiences of body-shaming related to the use of TikTok, avoidance of unpleasant contents, and possible effects on self-esteem. A 4-point Likert scale (never/seldom/sometimes/often) was used to collect data on the frequency for visualization of contents concerning pro-Ana/pro-Mia and Eating Disorders recovery/awareness (pro-ED recovery/pro-ED awareness). The same Likert was used to assess the frequency for content visualization of different categories (entertainment; dance; pranks; fitness/sports; home renovation/ “do it yourself”; beauty/skincare; fashion; recipes/cooking; life advices; pets; make-up; food and diets). A 5-point Likert scale was used to assess the subjective feeling of motivation related to the visualization of pro-Ana/pro-Mia contents (very low, low, unchanged, high, very high). The number of hours per day of TikTok use was assessed on an ordinal scale. Last, open-ended questions on searched hashtags (pro-Ana/pro-Mia and pro-ED recovery/pro-ED awarness) and the concurrent use of Instagram were included. Two independent physicians (L.C., A.Pe.) administered the survey to patients during routine evaluations for ED.

### Ethical aspects

Due to the anonymous nature of the survey, an approval from the Ethical Committee was not required. Each participant was informed of the scientific scope and anonymous nature of the questionnaire prior to its administration.

### Statistical analysis

Descriptive analyses were provided for demographic characteristics of the sample, variables concerning the use of TikTok (hours of use per day, frequency of interaction with subgroups of contents) and variables concerning the perceived effect of TikTok on the participants (self-esteem, changes in daily life, participation in challenges, victimization through body-shaming). Dichotomic variables concerning possible consequences of TikTok use (perceived negative effects on self-esteem and changes in daily life) were compared for the time spent using TikTok using Mann–Whitney U-test. Then, groups of patients with different perceived effects of TikTok were compared for the frequency of interaction with 3 specific contents (diet, pro-Ana, pro-ED recovery); for these multiple comparisons of measures belonging to the same domain, Bonferroni-corrected significance level was applied to the probability level (0.05/3 = 0.017). All statistical analyses were performed using JASP (Jeffreys's Statistics Program) v. 0.14.1 for Windows.

## Results

### Demographic characteristics of the sample

The examiners proposed the questionnaire to 91 patients with ED. The anonymous questionnaire did not ask to state the type of ED. Thirteen patients refused to be included in the survey for they did not wish to disclose personal information. No patient refused to join the survey due to a lack of use/insufficient knowledge of SM. Seventy-eight patients agreed to participate in the study and completed the questionnaire. Participants were 73 (93.6%) females and 5 (6.4%) males. The mean age was 14.5 ± 2.1 years. TikTok represented the main social media (SM) for 49 (62.8%) patients. Other preferred SM were Instagram (21.8%), YouTube (6.4%), WhatsApp (5.1%), Pinterest (2.6%), Facebook (1.3%), and the game Among Us (1.3%).

### Use of TikTok

The mean declared daily use of TikTok was 1.4 (± 1.0) hours. Frequencies of use of different subcategories of TikTok contents are reported in Table 1. Diet (21.8%), cuisine (20.5%) and dance (16.7%) represent the categories with the highest proportion of “often” use.

**Table 1:** Frequency of use of subcategories of TikTok contents

	Never	Seldom	Sometimes	Often	Does not answer—No use of TikTok
Entertainment	9.0%	30.8%	40.0%	9.0%	11.2%
Educational	26.9%	37.2%	20.5%	5.1%	10.3%
Fashion	9.0%	32.0%	35.9%	12.8%	10.3%
Diet	16.7%	19.2%	32.4%	21.8%	10.3%
Dance	26.9%	19.2%	25.7%	16.7%	11.5%
Sports	24.3%	24.3%	32.1%	9.0%	10.3%
DIY	43.6%	26.9%	17.9%	1.3%	10.3%
Skincare	24.4%	21.8%	33.3%	7.7%	12.8%
Cuisine	21.8%	16.6%	30.8%	20.5%	10.3%
Animals	43.6%	30.7%	10.3%	5.1%	10.3%
Makeup	30.8%	24.3%	23.0%	11.6%	10.3%

*Abbreviation*: *DIY* Do-it-yourself

### Perceived effects of TikTok

Forty-six patients (59.0%) reported that using TikTok and viewing its contents made them feel somewhat more insecure. Twenty-one patients (26.9%) reported significant changes in their daily lives due to the use of TikTok. Ten patients (12.8%) reported participation in at least one “challenge” related to the use of the SM. Lastly, 3 patients (3.8%) described that they were victims of episodes of body-shaming while using the SM.

### Pro-Ana and pro-ED recovery contents

**Fig. 1:**
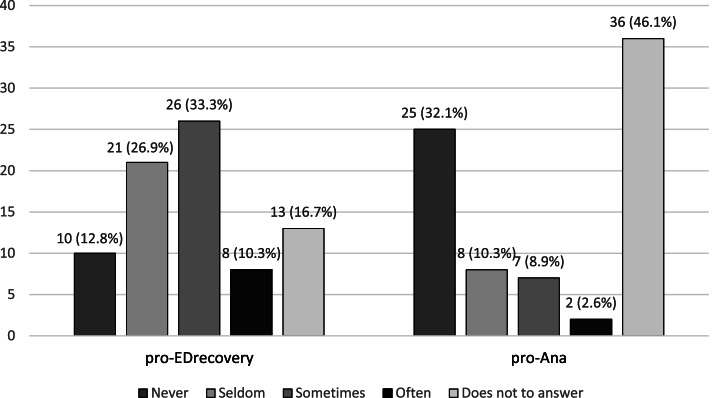
Frequency of active searching of pro-ED recovery and pro-Ana contents. Abbreviations: pro-Ana: pro Anorexia Nervosa; pro-ED recovery: pro eating disorders recovery

The frequency of active searching for pro-ED recovery and pro-Ana contents is reported in Fig. 1. Beyond active searching, the participants also reported the frequency by which they received unsolicited ED-related contents when using the SM: 50 individuals (64.1%) stated that they were proposed pro-ED recovery contents; 43 (55.1%) reported that they were proposed pro-Ana contents.

### Contents viewed and perceived effect of TikTok

Patients, who reported longer periods spent each day using TikTok, more frequently described a negative effect of TikTok on their self-esteem (*p* = 0.025). The hours per day spent on the applications were not different in patients reporting/not reporting changes in their daily lives due to TikTok (*p* = 0.155), or in patients participating/not participating in TikTok challenges (*p* = 0.207).

Patients reporting changes in their daily lives due to TikTok described a higher frequency of searches for pro-ED recovery contents (*p* = 0.015), while no significant difference was found as regards the frequency of searches for pro-Ana (*p* = 0.196) or “diet” (*p* = 0.024) contents. Patients reporting that they had participated in TikTok challenges were found to describe a more frequent search for “diet” contents (*p* = 0.005), while no significant differences emerged concerning their search for pro-ED recovery (*p* = 0.036) or pro-Ana (*p* = 0.456) contents. As for the patients describing a negative effect of TikTok on their self-esteem, a more frequent search for “diet” (*p* = 0.007) and pro-ED recovery (*p* = 0.007) contents was found; no differences emerged in the frequency of searches for pro-Ana contents between these patients and those who did not report a negative effect in their lives (*p* = 0.149).

### Hashtags and users’ comments

Pro-ED recovery and pro-Ana subgroups of frequently used hashtags are reported in Table 2. The most frequently used pro-ED recovery hashtag was “#edrecovery” (25 patients, 32.0%). The most frequently used pro-Ana hashtag were #foryou (6 cases, 7.7%) and #ed (5 cases, 6.4%). Participants’ comments on TikTok’s specific features are reported in Table 3. The most frequently cited positive feature concerned the nature of TikTok algorithm, connecting people with similar interests through the suggestion of specific contents (16 cases, 20.5%). The functioning of this algorithm represented the main negative feature of TikTok for 14 patients (17.9%), who reported repeated exposure to persuasive contents affecting their thoughts and behaviors.

**Table 2:** Pro-Ana and pro-ED recovery hashtags

Pro-ED recovery contents	N	Pro-Ana contents	N
**ED related hashtags**: #Ed #dca #eatingdisorder #disturbialimentari #anoressia #anorexia #ana #noproana #anoressiaitalia #bulimia #bingeeating	37 (47.4%)	**Generic/not strictly ED related hashtags**: #foryoupage #foryou #perte #virale #tw #challenge #dance #ironia #mentalillness #dieta #forma #sport	16 (20.5%)
**Recovery related hashtags**: #recovery #dcarecovery #edrecovery #anarecovery #anorexiarecovery #bingeeatingrecovery #whatieatinadayrecovery	28 (35.9%)	**ED related hashtags**: #ed #dca #proana #anoressia #anorexia #anoressiaitalia	10 (12.8%)
**Generic/not strictly ED related hashtags**: #foryou #perte #trend #tiktok #vita #salute #benessere #amore #body #foodlove #cibo #alimentazione #selflove #amati	16 (20.5%)	**Motivational eating routine related hashtags**: #whatieatinaday #whateatinaday #quellochemangioinungiorno #quantecalorie #fearfood #balanceddiet	8 (10.3%)
**Motivational related hashtags**: #dcafighter #anorexiafighter #nevergiveup #celapuoifare #vinciamoquestalotta #eatingmotivation #eatwithme #noricadutaanoressia #edhumor	10 (12.8%)	**Motivational lose weight/thin hashtags**: #workoutwithme #thinspo #thynspiration #fastloseweight #fasting #purging	6 (7.7%)
**Mental health related hashtags**: #mentalillness #mentalhealthmatters #mentalbreakdown	3 (3.8%)		

*Abbreviations*: *pro-Ana *pro Anorexia Nervosa; ED: eating disorders

**Table 3:** Participants’ comments on TikTok’s distinctive features

Positive comments	N	Negative comments	N
The algorithm shows you videos related to your interests, bringing similar people together, encouraging people to express themselves freely	16 (20.5%)	The algorithm offers the same trends over and over (“foryou” page), changing someone’s thoughts and behaviors	14 (17.9%)
Various, original, educational contents	11 (14.1%)	Lots of haters; body-shaming comments	10 (12.8%)
More space to mental health, with some specialists giving advice	8 (10.3%)	Many videos on physical appearance, leading to comparisons. [TikTok] influences beauty standards. People pretend to be someone that they are not	9 (11.5%)
More positive messages and less space for body-shaming	5 (6.4%)	Contents about mental health can be triggering, sometimes wrong advice is given	7 (9.0%)
Users are less afraid of showing themselves as they really are. More videos than pictures, so it is difficult to modify someone’s image	5 (6.4%)	More addictive than other SM	5 (6.4%)
More people of the same age, with more in common to share	4 (5.1%)	Challenges can be dangerous	4 (5.1%)
People may encourage you to eat and make videos to raise awareness about Eating Disorders	1 (1.3%)	Easy for children, under the age limit, to go on TikTok	2 (2.6%)

## Discussion

This is the first study to investigate the use of specific TikTok contents in a sample of patients with ED in developmental age. From the analysis of the results, it is possible to note how among the patients between 12 and 16 years of age (the average age of our sample was 14 and a half years), TikTok represents the main SM, particularly among the younger patients (62.8%). This underlines its importance as a means to communicate and disseminate content. This result should be compared with the analyses conducted by Vall-Roqué and colleagues [39] on a sample of 2601 women living in Spain. The subjects included in this study were divided into generation Z (aged from 14 to 24) and generation Y/Millennials (between 25 and 35 years old). The authors found that participants from generation Z used Instagram, YouTube, TikTok and Twitter more frequently, whereas participants from Generation Y used Facebook more often.

In this same study, significant relationships emerged between the frequency of Instagram use and body dissatisfaction, drive for thinness and low self-esteem in adolescents and young adults (14–24 years), and between the frequency of Instagram use and drive for thinness in the older age group (25–35 years). Thus, continuing modifications in the use of SM by children and adolescents may potentially lead to the development of disordered eating cognition and eating disorders in new generations.

In our study, the most researched categories are represented by “Diet” and “Cuisine”. A recent study by Pilar and colleagues [40] examined the diet topic on Instagram. The authors analyzed 2,045,653 messages created by 427,936 individual users, showing that users mostly associate healthy food with healthy lifestyle, diet, weight loss, and fitness. The authors concluded that, since young people may change their behavior in relation to others’ behavior on SM, these data could help predict their future association with healthy food characteristics. Since our sample has been selected from a population referring to a third level Center for ED, we believe our results should not be directly compared with data collected from the general population, and should be verified in the future through specific studies of young people with ED.

Significantly, 64% of the patients in our sample declared that they found pro-Ana materials on TikTok’s bulletin board without actively searching. This result should encourage the preparation of specific studies further investigating the relationship between SM algorithms and the spread of disordered eating behaviors among children and adolescents.

A recent cross-sectional study conducted in Italy on patients with ED [13] documented that more than 80% of the respondents used Internet daily. From 2017 to 2020, the authors observed a rise of the pro-Ana phenomenon among adolescents. They reported that in 2020, 76% of the patients knew of sites emphasizing a marked thinness as an ideal of beauty, 60% knew of explicitly pro-Ana sites, and 22% visited them. The presence of pro-Ana contents on Twitter has been recently studied by Sukunesan and colleagues [41]. The authors concluded that pro-ED online communities are transient in nature as they engage in superficial discussion threads, but they are also resilient, emulating cybersectarian behaviors. Moreover, as documented by a recent case report on the use of TikTok, even “anti-pro-Ana” videos may paradoxically lead users to emulate ED-related behaviors and non-suicidal self-injury [42].

However, the use of TikTok may account for specific differences from other SM concerning the process of selection of virtual contents. Interestingly, a recent research assessed suicide incidence among adolescents and youths during the COVID-19 pandemic [43]. This study showed that TikTok addiction-related psychological distress represented one of the main suicide stressors, following mental sufferings and online schooling. Thus, clinicians and researchers should address specific concerns to properly identify possible risks originating from the use of TikTok by young people with mental illnesses and ED. We want to emphasize that our study was conducted during the second Italian national lockdown due to the COVID-19 pandemic. This may have influenced the risk factors behind the development of ED in our patients. Restrictions due to the pandemic could also have altered the way our patients used SM, by increasing the hours per day spent on TikTok and expanding the interaction with potentially problematic contents.

In our sample, 59% of the investigated patients said that they felt more insecure after viewing contents related to diet and food, while a quarter of them deliberately changed their habits after viewing them. The negative effect on self-esteem was correlated with the average daily time of use of the social network. Our data expand the results of a recent study [44], which documents an association between SM usage and disordered-eating cognitions and behaviors occurring at a younger age than previously found. Despite these results, we do not think that the use of TikTok should be demonized or discouraged altogether, but it could be allowed or encouraged, for example, with parental control. Moreover, the positive and negative feedback that patients expressed qualitatively should be valued at least as much as statistic data are. These results may support further studies directly addressing the potential effect of extended usage of SM on self-esteem in young individuals with ED and other mental health conditions.

A relevant difference between the use of pro-ED recovery and pro-Ana contents emerges from statistics concerning unanswered questions. That is, the question concerning the active search of ED-related contents remained unanswered in 13 cases (16.7%) for pro-ED recovery, and in 36 cases (46.2%) for pro-Ana. The high frequency of lack of answers for active searches of pro-Ana contents stands out in the survey. An indirect explanation could be that some patients did not answer because they preferred not to disclose personal information to physicians or their parents. As a recent study on impulsivity traits and early maladaptive schemas in individuals with Anorexia Nervosa (AN) document, restraint may represent a distinguishing feature in AN patients [45]. People with AN, and with ED in general, may be exposed to stigmatizing attitudes and beliefs, as reported in the context of communities of undergraduate students [46] and healthcare students [47]. Hence, the possible effect of restraint and fear for stigma should be considered in the interpretation of our results.

In addition to relaying comments on TikTok’s negative features, a relevant number of individuals in our sample mentioned the use of hashtags promoting ED-recovery, with 32.0% of the respondents reporting the use of “#edrecovery”. This positive use of SM has been specifically addressed in a recent study by Herrick and colleagues [36]. The authors conducted a systematic review and inductive thematic analysis of 150 TikTok posts compiled under the hashtag “#edrecovery”. Five themes were identified across the data: (a) ED awareness, (b) inpatient story time: “ED unit tings”, (c) eating in recovery, (d) transformations: “how about a weight gain glow up?”, and (e) trendy gallows humor: “let’s confuse people who have a good relationship with food”. The researchers concluded that TikTok may represent an artistic and social tool for some individuals to share their thinking about the ED recovery narrative and promote a semblance of community. Given the conflicting meanings and effects of a series of hashtags and contents related to ED on TikTok, the literature needs further studies focusing on the impact of the hashtag and other features of SM on individuals with ED, and their potential use in the management of these conditions.

We acknowledge the main limitations in our study: the sample considered for this research was made up almost entirely by females; moreover, our population included patients treated in an eating disorder clinic. Hence, our study lacks comparative data with a control group. The enrollment process may have selected individuals positively disposed to disclose personal information on the use of TikTok and other SM. The assessment of those who refused to participate could have revealed specific motivations and potential concerns about stigmatizing attitudes toward their personal life, as possibly indicated by the high number of unanswered questions on active searches for pro-Ana contents.

However, this study also presents significant strengths, for it assesses a homogeneous group of individuals who were all evaluated at a third-level Center, and documents the use of a specific social media in a younger and scarcely investigated age group during the COVID-19 pandemic.

## Conclusion

Our research documents the pattern of TikTok use in a sample of 78 children and adolescents with ED. TikTok represented the most used SM for the majority of the individuals in the sample. Participants shared both positive and negative perspectives on the use of this SM platform. Further studies are needed to systematically analyze the relationship between SM and the pathogenesis and treatment options for ED.

## Data Availability

The datasets used and analyzed during the current study are available from the corresponding author upon reasonable request.
